# Pancreatic cancer cell exosomes induce lipidomics changes in adipocytes

**DOI:** 10.1080/21623945.2022.2084900

**Published:** 2022-06-23

**Authors:** Shihua Wang, Meiqian Xu, Xian Xiao, Liping Wang, Zhao Sun, Mei Guan, Robert Chunhua Zhao

**Affiliations:** a Institute of Basic Medical Sciences Chinese Academy of Medical Sciences, School of Basic Medicine Peking Union Medical College, Center of Excellence in Tissue Engineering, Chinese Academy of Medical Sciences; Beijing Key Laboratory of New Drug Development and Clinical Trial of Stem Cell Therapy (BZ0381); b Department of Otolaryngology-Head and Neck Surgery, Laboratory of ENT-HNS Disease, First Affiliated Hospital of Guangzhou Medical University, Guangzhou, China; c Department of Oncology, Peking Union Medical College Hospital, Chinese Academy of Medical Sciences and Peking Union Medical College, Beijing, China; d Department of Cell Biology, School of Life Sciences, Shanghai University, Shanghai, China

**Keywords:** Exosome, adipocyte, pancreatic cancer, lipolysis, IL-6

## Abstract

Increasing evidence has demonstrated the important roles of exosomes during pancreatic cancer development. However, the effects of pancreatic cancer exosomes (PC-exos) on adipocytes remain largely unknown. Here, we used mass-spectrometry-based lipidomics to identify lipids that were changed in adipocytes after exposure to PC-exos, and we found that triglyceride (TG) reduction was the most significant, which might be induced by increased lipolysis because the number of large lipid droplets increased while small ones decreased. Additionally, abdominal adipocytes in mice injected with PC-exos had a relatively smaller size. Mechanistically, we found that genes involved in metabolism and inflammation were up-regulated, among which increase of IL-6 was significant, and we then found IL-6 promoted lipolysis. To our knowledge, this is the first study on the lipidomics changes of adipocytes after PC-exos treatment.

## Introduction

Despite significant improvements in therapeutic strategies, pancreatic cancer (PC) remains one of the lethal malignancies with rising incidence worldwide [1]. One key pathological characteristic of PC is that PC has a highly desmoplastic reaction, suggesting an intense tumour cells and stroma communication [2]. The tumour stroma contains a heterogeneous population of cells, including cancer-associated fibroblasts (CAFs), endothelial cells, immune cells, and adipocytes. It has been reported that tumour cells could ‘educate’ these cells by transferring information via exosomes to promote the formation of favourable tumour microenvironment [3,4]. Exosomes are now widely recognized as key players in intercellular crosstalk between cancer cells and other cells through horizontal transfer of their cargos, which includes proteins, lipids, and nucleic acids [5]. Costa-Silva et al. found that exosomes derived from malignant pancreatic lesions promoted liver metastasis by supporting pre-metastatic niche formation through a multi-step process involving the education of liver Kupffer cells to generate a fibrotic microenvironment [6]. Zhe et al. showed pancreatic cancer-initiating cell exosomes promoted v6kd and Tsp8kd non-cancer-initiating cell activation, apoptosis-resistance, EMT, motility and tumour progression [7]. Another recent study reported that PC exosomes could be taken up by T lymphocytes to activate p38 MAPK, and then induce ER stress-mediated apoptosis, ultimately causing immunosuppression [8]. Increasing evidence is beginning to reveal the important roles of exosomes in a variety of pathological processes during PC development. However, the effects of PC exosomes on adipocytes, a critically important component of the adipose tissue, remain largely unexplored.

Currently, only a few studies have reported the effects of tumour exosomes on adipocytes. Wu et al. found that breast cancer exosomes promoted tumour progression through the promotion of beige/brown differentiation and the increase in catabolism in adipocytes [9]. We have shown that hepatocarcinoma-cell-derived exosomes could endow adipocytes with tumour promoting effects [10]. However, to our knowledge, so far, only one study investigated the effects of PC exosomes on adipocytes. It reported that PC exosomes promoted lipolysis in both murine and human adipocytes and activated mitogen-activated protein kinases [11]. Here, we further explored the effects of PC exosomes on the lipidomics changes of adipocytes and identified 190 lipids with significant changes after exposure to PC exosomes, among which the down-regulation of triglyceride (TG) is remarkable. The reduced levels of TG might be caused by increased lipolysis. PC exosomes could significantly decrease the formation of large lipid droplets and increase the number of small lipid droplets. Moreover, in mice injected with PC exosomes, adipocytes from abdominal adipose tissue had reduced size. Mechanistically, we found that genes involved in metabolism and inflammation were up-regulated, among which increase of IL-6 was significant, and we then found IL-6 promoted lipolysis. To our knowledge, this is the first study on the lipidomics changes of adipocytes after PC exosomes treatment, which may provide new insights into understanding the interactions between tumour cells and adipocytes.

## Materials and methods

### Cell culture

Human adipose tissues were obtained according to procedures approved by the Ethics Committee at the Chinese Academy of Medical Sciences and Peking Union Medical College. MSCs were isolated and culture-expanded from healthy donors as reported before [12]. MSCs of passage 3–5 were used for adipogenic differentiation. The contents of adipogenic induction medium and the procedures were the same as that described in our published work [9]. Adipocytes were characterized by Oil red O staining and Bodipy staining according to manufacturers’ instructions. Pancreatic cancer cell lines AsPC-1 and PANC-1 were purchased from cell bank at the Chinese Academy of Medical Sciences and cultured in DMEM containing 10% FBS, penicillin (100 U/mL) and streptomycin (100lg/mL) at 37°Cwith 5% CO_2_.

### Exosome isolation and characterization

Exosomes were isolated from cell conditioned medium using ultracentrifugation method as previously described [10] and were quantified by BCA protein quantification.

### Exosome labelling

Exosomes were labelled with PKH26 and added into the culture medium of adipocytes for 1 h, 6 h, and 12 h. The cell nucleus was labelled with Hochest 33342 (1:500, Sigma-Aldrich). Internalization of exosomes by adipocytes was then visualized by fluorescent microscopy.

### Mass-spectrometry-based lipidomics

Mass-spectrometry-based lipidomics was performed by Shanghai Sensichip Hightech Co., Ltd. (Shanghai, China). Briefly, adipocytes in chloroform/water/methanol (2/1/1, v/v/v) solution were vortexed, centrifuged at 2500 rpm for 15 min, and then the organic phage was collected and lyophilized using nitrogen. After lipid extraction, reversed-phase analysis of lipids was performed on the Dionex Ultimate 3000 High-Performance Liquid Chromatography (HPLC) system coupled to an Orbitrap Elite in profile mode (Thermo Fisher Scientific, USA). Parameters used for mass spectrometry were described as follows: positive ion mode, Heater Temp-300°C, Sheath Gas Flow rate-45arb, Aux Gas Flow Rate-15 arb, Sweep Gas Flow Rate-1 arb, spray voltage-3.0 kV, Capillary Temp-350°C, S-Lens RF Level-30%. Scan ranges: 200–1500; negative ion mode, Heater Temp-300°C, Sheath Gas Flow rate-45 arb, Aux Gas Flow Rate-15 arb, Sweep Gas Flow Rate-1 arb, spray voltage- 2.5 kV, Capillary Temp-350°C, S-Lens RF Level-60%. Scan ranges: 200–1500. Raw data were first processed by the Thermo SIEVE 2.1 Qualitative Analysis Software

(Thermo Fisher Scientific, USA) and then normalized to the total area. All data with peak numbers (based on the retention time and mass-to-charge ratio (m/z)) were imported into SIMCA-P + 13.0 (Umetrics, Sweden).

### Glycerol release

Glycerol release into the cultured media was determined using free glycerol determination kit (Sigma-Aldrich) under manufacturer’s instructions. A glycerol standard curve ran alongside each assay and glycerol release was quantified on a microplate reader at a wavelength of 570 nm.

### Quantitative real-time polymerase chain reaction(qRT-PCR)

Total RNA was extracted using TRIzol (Invitrogen) according to the manufacturer’s instruction, and cDNA was prepared. Real-Time PCR amplification was performed in triplicates according to procedures reported previously [13]. Relative expression of mRNA was evaluated by 2-ΔΔCt method and normalized to the expression of GAPDH.

### Mice experiments

BALB/C mice (6–8 weeks) were purchased from the Laboratory Animal Center of the Chinese Academy of Medical Sciences (Beijing, China). All mice were bred and maintained under specific pathogen-free conditions. Animal use and experimental procedures were approved by the Animal Care and Use Committee of the Chinese Academy of Medical Sciences. The mice were randomly divided into three groups and intraperitoneally injected with 200ug AsPC-1 exosomes, PANC-1 exosomes and PBS, respectively, every 3 days for three times. The mice were sacrificed 3 days after the last operation. Abdominal and mesenteric adipose tissues were harvested and fixed with 10% PFA. HE staining was used to detect the size of the adipocytes.

### Statistical analysis

Statistical analyses of the data were performed by Prism 6.0 (GraphPad Software Inc.) using one-way ANOVA. Differences were considered statistically significant at *P < 0.05, **P < 0.01 and ***P < 0.001.

## Results

### Adipocytes uptake exosomes derived from pancreatic cancer cell lines AsPC-1 and PANC-1

**Figure 1.**
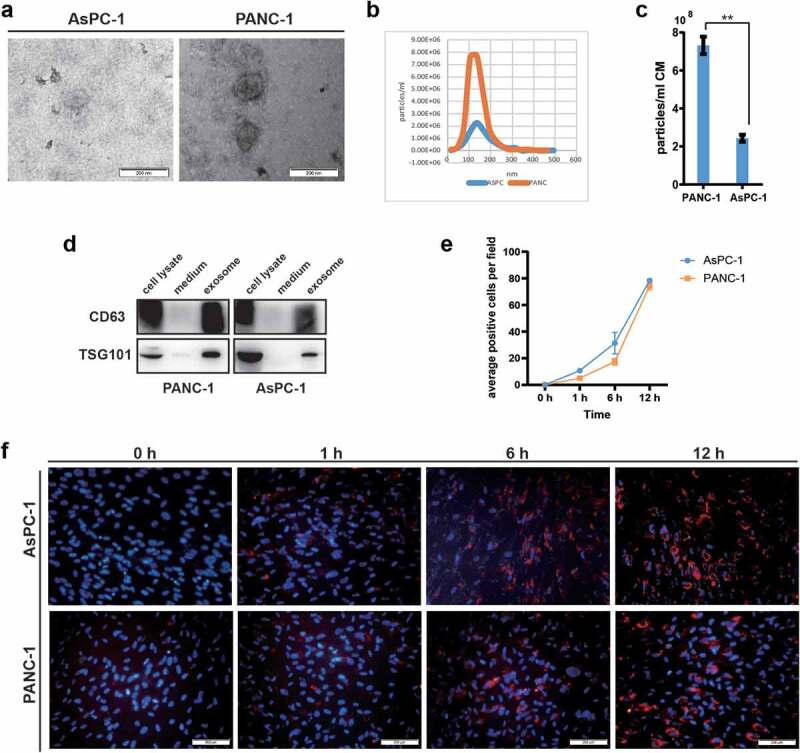
Adipocytes incorporated exosomes derived from pancreatic cancer cells AsPC-1 and PANC-1.

We isolated exosomes from the conditioned medium of pancreatic cancer cell lines AsPC-1 and PANC-1 using ultracentrifugation method and characterized them by TEM, NTA and western blot. As shown in Figure 1(a–c), exosomes had a round spherical or cup-shaped ultrastructure with diameters ranging from 30 to 200 nm and PANC-1 cells secreted more exosomes compared to AsPC-1. Western blot analysis showed that these exosomes were positive for classic exosome markers including CD63 and TSG101 (Figure 1(d)).

(a) A representative electron microscopy image of AsPC-1 and PANC-1 exosomes. (b) The nanoparticle size distribution for AsPC-1 and PANC-1 exosomes detected by NTA. (c) PANC-1 secreted more exosomes as compared to AsPC-1. (d) Western blot analysis of exosome marker CD63 and TSG101. (E, F) Adipocytes were incubated with 200 μg/mL PKH26-labelled exosomes for the indicated times, and the uptake of exosomes was determined by fluorescence microscopy. E was the quantification of exosome internalization; F was the representative images (scale bar, 200 μm).

We have previously demonstrated that human in vitro differentiated adipocytes could serve as a good *in-vitro* cellular model for adipocytes research [14]. The differentiated adipocytes had lipid droplets in cytoplasm as shown by both BODIPY and Oil Red O staining (Supplementary Figure. S1A). Marker genes for adipocytes such as Adipo Q, c/EBPα, LPL, HSL, FABP4, aP2, and PPARγ were also significantly up-regulated during adipocyte induction as measured by qRT-PCR (Supplementary Figure. S1B).

Successful entry into target cells of tumour exosomes is a prerequisite for tumour cells to reprogram or remodel surrounding microenvironment [15]. Here, we investigated adipocyte internalization of pancreatic cancer exosomes using the membrane dye Dil fluorescent labelled exosomes. Confocal images showed obvious evidence of the cellular uptake of pancreatic cancer exosomes by adipocytes as early as 1 h after co-culture and peaked at 12 h (Figure 1(e,f)). Taken together, these results show that pancreatic cancer cells secrete exosomes with typical exosomal features and are actively taken up by adipocytes.

### Pancreatic cancer cell exosomes disrupt the lipid composition of adipocytes, particularly the TG species

**Figure 2.**
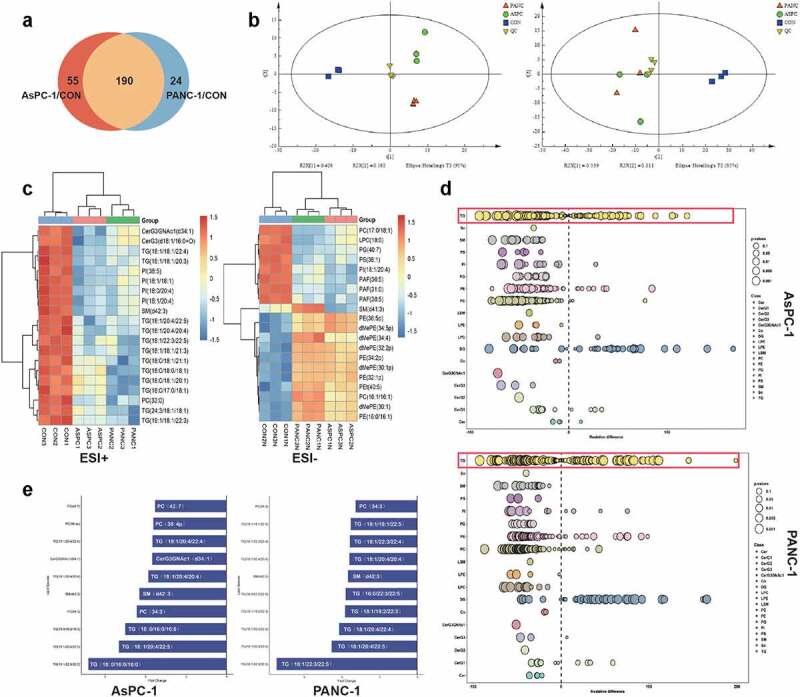
PANC-1 and AsPC-1 exosomes disrupted the lipid composition of adipocytes, particularly the TAG species.

To investigate the lipid composition changes in adipocytes in response to pancreatic cancer cell exosomes, we applied mass-spectrometry-based lipidomics. A total of 245 and 214 lipids were significantly changed after exposure to PANC-1 and AsPC-1 exosomes, respectively (fold change >2 or <0.5 in exosome-treated adipocytes vs control adipocytes). One hundred and ninety lipids were identified as jointly changed by both PANC-1 and AsPC-1 exosomes (Figure 2(a)). PCA analysis demonstrated a clear separation of adipocytes treated by exosome from control adipocytes (Figure 2(b)), suggesting great changes of lipidomics in adipocytes induced by pancreatic cancer cell exosomes. Further analysis revealed the top 20 significantly differentially expressed lipids, among which a remarkable down-regulation in triglyceride (TG) was observed (Figure 2(c)). TG storage in adipocytes provides the major reservoir for metabolic energy [16]. Considering the importance of this lipid species, we focused on it in our following experiment. We further analysed the relative percentage differences in concentrations of all quantified lipid species between PANC-1 or AsPC-1 exosomes treated adipocytes and control adipocytes (Figure 2(d)). We also used multivariate statistical analysis to identify the top 10 differential lipid substances (Figure 2(e)). All these results suggested the significant downregulation of TG in adipocytes after treatment with pancreatic cancer exosomes.

(a) A Venn diagram showed the number of lipids significantly changed (fold change >2 or <0.5 in exosome-treated adipocytes vs control adipocytes) after exposure to PANC-1 or AsPC-1 exosomes. (b) The PCA scores plot of all samples; the left panel was calculated under ESI+ mode, the right panel was ESI-mode. (ESI is electrospray ionization, under ESI+ mode, molecules with positive ion signal could be detected, while under ESI-mode, molecules with negative ion signal could be detected). (c) The heat map representing the differential abundance of the top 20 selected lipids between exosome treatment groups and control groups (the left was ESI+ mode, the right panel was ESI-mode). (d) The relative percentage difference in concentration of all quantified lipid species between PANC-1 (left panel) or AsPC-1 exosomes (right panel) treated adipocytes and control adipocytes. Each dot represents a lipid species, and the dot size indicates significance. (e) Top 10 altered lipids in adipocytes following treatment with PANC-1 exosomes (left panel) and AsPC-1 exosomes (right panel).

### Pancreatic cancer exosomes stimulate lipolysis in adipocytes both in vitro and in vivo

**Figure 3.**
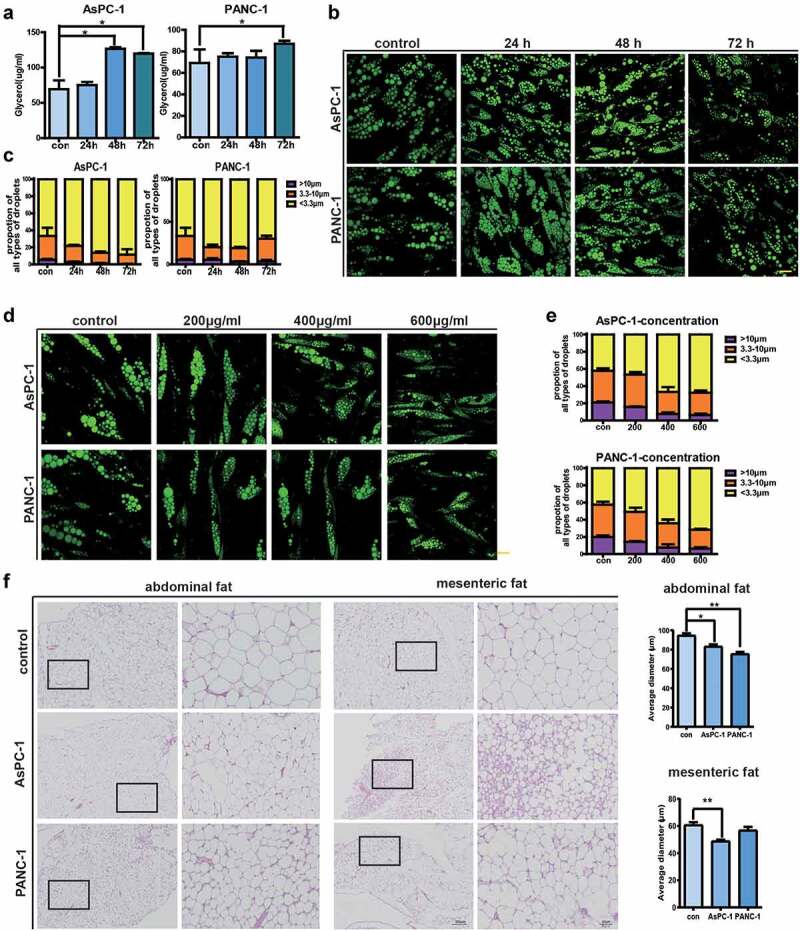
Pancreatic cancer exosomes stimulate lipolysis in adipocytes both *in vitro* and *in vivo.*

Under physiological conditions, the level of TG in adipocytes is tightly regulated by the delicate balance between TAG synthesis (lipogenesis) and breakdown (lipolysis) [17]. Reduced levels of TG after exposure to pancreatic cancer exosomes prompted us to investigate adipocyte lipolysis, which leads to the breakdown of TG into glycerol and fatty acids [16]. We first confirmed that glycerol release was increased in adipocytes after treatment with PANC-1 and AsPC-1 exosomes (Figure 3(a)). We then examined lipid droplets (LDs) in adipocytes which are critical for the handling of lipolysis. We categorized LDs into three types, with diameters >10 μm as Large LD, diameters 3.3–10 μm as Middle LD, and diameters <3.3 μm as Small LD. Fluorescence microscopy showed that PANC-1 or AsPC-1 exosomes treatment reduced the formation of Large LDs (percentage of LDs was 1.68 ± 0.19% in PANC-1 exosome-treated adipocytes VS 6.73 ± 0.65% in control adipocytes; 0.33 ± 0.12% in AsPC-1 exosome-treated adipocytes VS 5.23 ± 0.97% in control adipocytes) and increased the number of Small LD (percentage of Small LDs was 72.78 ± 2.66% in PANC-1 exosome-treated adipocytes VS 60.39 ± 9.17% in control adipocytes; 88.53 ± 8.16% in AsPC-1 exosome-treated adipocytes VS 66.63 ± 14.39% in control adipocytes) (Figure 3(b,c)). Moreover, this effect is a bit of concentration-dependent. When exposed to 600 μg/ml pancreatic cancer exosomes, the number of large LDs significantly reduced, while the number of small LDs increased (Figure 3(d,e)). To further investigate the effect of pancreatic cancer exosomes on adipocyte lipolysis, we intraperitoneal injected PANC-1 or AsPC-1 exosomes into mice and analysed the sizes of adipocytes 2 weeks later. No obvious alterations in weight loss were observed. However, as shown in figure 3(f), in mice injected with AsPC-1 exosomes, adipocytes from both abdominal and mesenteric adipose tissue had reduced size with statistical significance, and in mice injected with PANC-1 exosomes, adipocytes from abdominal adipose tissue had reduced size compared with control mice. Together, these data suggest that pancreatic cancer exosomes could promote lipolysis in adipocytes both in vitro and in vivo.

(a) glycerol release in adipocytes after treatment with PANC-1 or AsPC-1 exosomes for 24 h,48 h, and 72 h. (b) adipocytes were treated with 200 μg/ml PANC-1 or AsPC-1 exosomes for 24 h,48 h, and 72 h, then the LDs were stained with BODIPY 493/503 and visualized using a confocal microscope. Scale bar = 10 μm. (c) LD size was determined by measuring the diameter of at least 300 LDs using the ImageJ software. (d) Adipocytes were treated with different concentrations of PANC-1 and AsPC-1 exosomes for 72 h, then the LDs were stained with BODIPY 493/503 and visualized using a confocal microscope. Scale bar = 10 μm. (e) LD size was determined by measuring the diameter of at least 300 LDs using the ImageJ software. (f) H&E staining of abdominal and mesenteric adipose tissue in mice injected with PANC-1 or AsPC-1 exosomes. Scale bar, 200 μm, and for enlarged image, Scale bar, 50 μm.

### Transcriptomic analysis identifies expression changes of genes involved in metabolism and inflammation

**Figure 4.**
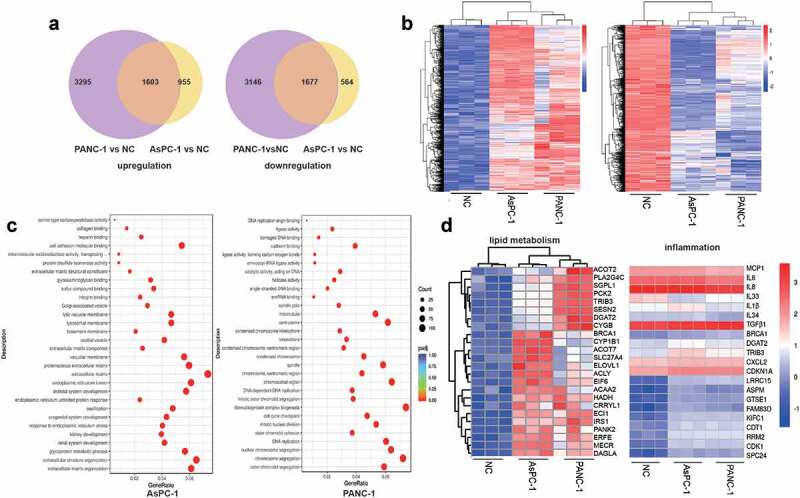
Transcriptomic analysis identifies expression changes of genes involved in metabolism and inflammation.

To further investigate the effect of PC exosomes on adipocytes, we analysed the transcriptomic profiles of adipocytes exposed to PANC-1 or AsPC-1 exosomes for 24 h. 4898 genes were up-regulated, 2558 genes were down-regulated after treatment with PANC-1 exosomes while 4823 genes were up-regulated, 2241 genes were down-regulated after treatment with AsPC-1 exosomes, among which 1603 genes were the commonly upregulated and 1677 were commonly downregulated genes by both PANC-1 and AsPC-1 exosomes (Figure 4(a,b)). We then performed a gene ontology analysis on these genes (Figure 4(c)) and specifically found that genes associated with lipid metabolism and inflammation were up regulated (Figure 4(d)).

(a) A Venn diagram showed the number of genes that was up- or down-regulated by PANC-1 or AsPC-1 exosomes. (b) Heat map showed differentially expressed genes in PC exosome-treated adipocytes and control adipocytes. (c) GO analysis of genes that were up- or down-regulated by PANC-1 or AsPC-1 exosomes. (d) Unsupervised hierarchical clustering based on gene expression associated with lipid metabolism and inflammation.

### Pancreatic cancer exosomes increase IL-6 production which in turn promotes lipolysis in adipocytes

**Figure 5.**
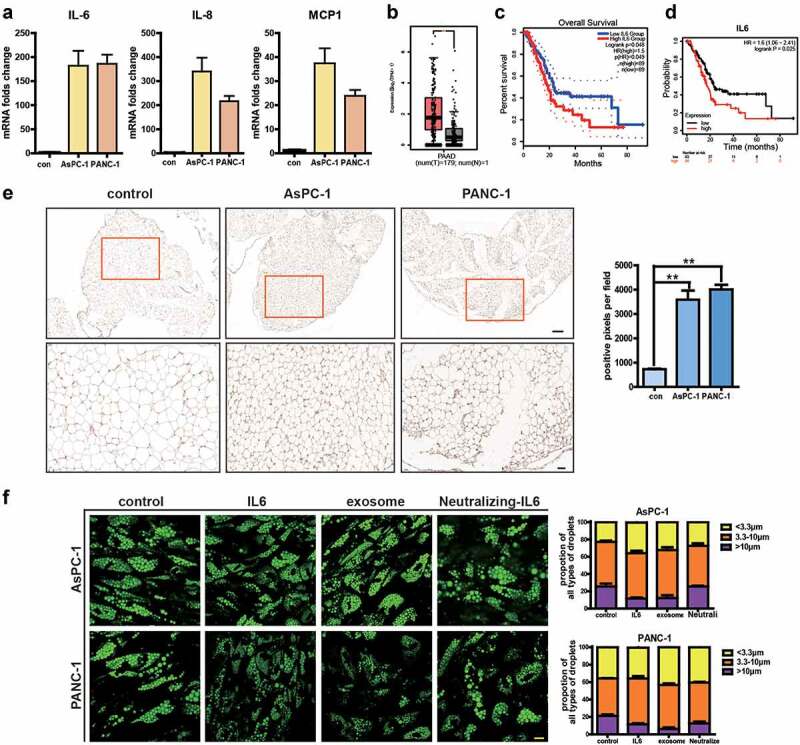
Pancreatic cancer exosomes increase IL-6 production which in turn promotes lipolysis in adipocytes.

Expression changes in genes associated with inflammation prompted us to further investigate the release of inflammatory cytokines in adipocytes. Enhanced release of IL-6, IL-8, and MCP-1 is shown in Figure 5(a). It has been demonstrated that IL-6 is instrumental for the maintenance and progression of pancreatic cancer and lack of IL-6 completely ablated cancer progression, even with activation of oncogenic Kras [18,19]. We first used GEPIA2 database (http://gepia2.cancer‐pku.cn/), a web‐based tool for gene expression on TCGA and GTExdata, to analyse the expression of IL-6 in pancreatic adenocarcinoma (PAAD) and found a higher expression of IL-6 in PC (Figure 5(b)). Higher IL-6 expression is correlated with poor prognosis as analysed by both Kaplan‐Meier Plotter (Figure 5(c)) and GEPIA2 (Figure 5(d). Kaplan‐Meier Plotter database (http://kmplot.com/) is an online tool to rapidly analyse the effect of gene expression on survival in 21 cancer types. Additionally, we analysed the correlation between IL-6 levels and that of several adipocyte surrogate genes in PAAD and found IL-6 correlated with adipoQ, FABP4, HSL, LPL, and PLIN1, but not with CEBP and PPAR gamma based on GEPIA2 (Supplementary Figure S2). We then analysed IL-6 in adipose tissue of mice injected with AsPC-1 or PANC-1 exosomes and found higher expression of IL-6 after PC exosome treatment (Figure 5(e)). Next, we evaluated whether the lipolysis-promoting effects of PC exosomes were mediated by IL-6. Addition of IL-6 neutralizing antibody abolished PC exosomes’ effect, while IL-6 could increase lipolysis (figure 5(f)). These results suggested that PC exosomes may act through IL-6 production.

(a) Expression levels of IL-6, MCP1, IL8 were detected by qRT-PCR. (b) The expression levels of IL-6 in PAAD as analysed by GEPIA2 database. * Indicated that *P*‐value is <0.01. (c, d) The prognostic potential of IL-6 in PAAD was evaluated by Kaplan‐Meier Plotter (c) and GEPIA2 (d). (e) Immunohistostaining analysis of IL-6 expression in adipose tissue of mice injected with AsPC-1 or PANC-1 exosomes. (f) LDs were stained with BODIPY 493/503 and visualized using a confocal microscope. Scale bar = 10 μm. LD size was determined by measuring the diameter of at least 300 LDs using the ImageJ software.

### Discussion

Adipocytes are an important cellular component of the tumour microenvironment. They could exert their effects not only as a type of energy-storing cells but also as endocrine cells through secreting a variety of bioactive molecules such as hormones, growth factors, and adipokines [20,21]. In this study, we used human mesenchymal stem cell differentiated adipocytes as a cellular model which could better recapitulate features of human adipocytes compared with 3T3-L1. We have previously used this model to demonstrate the effects of HCC exosomes on phenotypic transformation of adipocytes and found adipocytes could be converted into tumour-promoting cells [10]. Here, we investigated the effects of PC exosomes on adipocytes, as the correlations between pancreatic cancer and adipocytes are very strong. Accumulating evidence have revealed that obesity increases the risk of pancreatic cancer development. For example, David et al. showed that a diet high in fats and calories lead to obesity and metabolic disturbances and accelerated early pancreatic neoplasia in the conditional KrasG12D mouse model [22]. Joao et al. also found that obesity promoted desmoplasia associated with accelerated pancreatic tumour growth and impaired delivery/efficacy of chemotherapeutics through reduced perfusion [23]. Most of the studies focused on the effects of adipocytes on tumour progression. Only a few studies have investigated the effects of tumour cells on adipocytes. When 3T3-L1 adipocytes were co-cultured with pancreatic cancer cells (PANC-1) using the transwell system, adipocytes changed morphologically to fibroblast-like cells [24]. The factors secreted by tumour cells are likely to be the key mediators regulating changes of adipocytes into cancer-associated adipocytes.

Exosomes are a novel way of cell–cell interaction. Tumour exosomes have been demonstrated to play a critical role in tumour initiation and progression by transporting cargos into target cells and then profoundly influence their phenotype and function [25]. Increasing evidence suggested that pancreatic tumour derived exosomes could educate surrounding cells in the tumour microenvironment or other cells in the distance to create a favourable milieu. For example, PDAC-derived exosomes could induce liver pre-metastatic niche formation in naive mice and consequently increase liver metastatic burden through targeting Kupffer cells and hepatic stellate cells [6]. Pancreatic cancer cells produced miR-301a-3p-rich exosomes in a hypoxic microenvironment, which then polarized macrophages to M2 to promote malignant behaviours of pancreatic cancer cells [26]. Jin et al. found that PC-1.0 (a highly malignant pancreatic cell line) cell-derived exosomes could be taken up by and enhance PC-1 (a moderately malignant pancreatic cell line) cell proliferation, migration, and invasion abilities [27]. However, until now, only one group has investigated the effects of PC exosomes on adipocytes. They found that PC exosomes promoted lipolysis in both murine and human adipocytes and activated mitogen-activated protein kinases [11]. Here, we investigated the effects of PC exosomes on adipocytes and found that (i) Pancreatic cancer exosomes could disrupt the lipid composition of adipocytes and a remarkable down-regulation in TG was observed; (ii) PC exosomes treatment significantly reduced the formation of Large LDs and increased the number of Small LD in adipocytes, and reduced adipocytes size after injection in mice, suggesting that PC exosomes could promote lipolysis in adipocytes both in vitro and in vivo; (iii) PC exosomes increased inflammatory factor IL-6 production which in turn promoted lipolysis in adipocytes. Our findings may provide new insights into our understanding of the interactions between tumour cells and adipocytes.

## Supplementary Material

Supplemental Material

## Data Availability

The datasets used and/or analysed during the current study are available from the corresponding author on reasonable request.
