# Supplementary figures and images for: Pancreatic cancer cell exosomes induce lipidomics changes in adipocytes
Source: Adipocyte. 2022 Jun 23;11(1):346–55. doi: 10.1080/21623945.2022.2084900 (PMC9235897; doi:10.1080/21623945.2022.2084900)

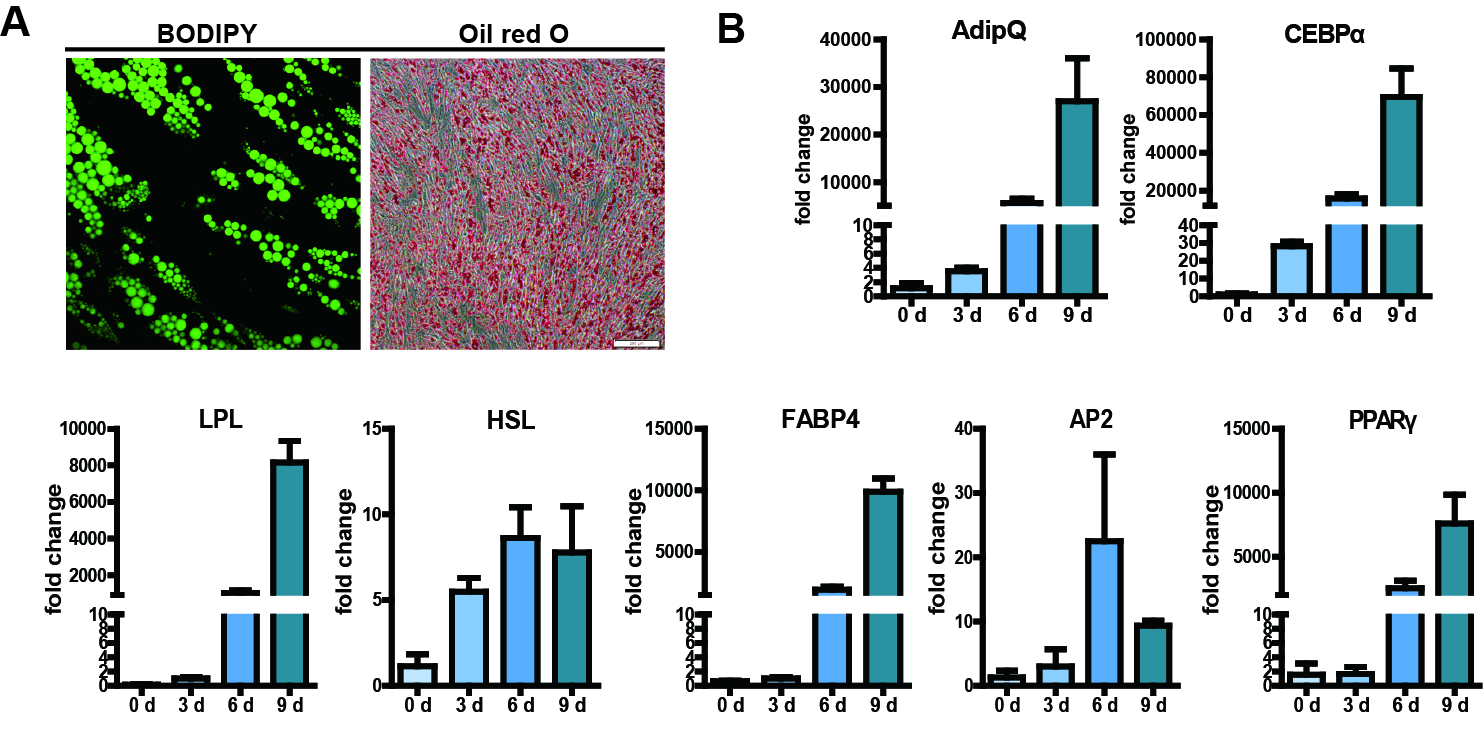

Supplement: Supplemental Material [file KADI_A_2084900_SM5599.zip › supplementary/Fgure S1.tif]

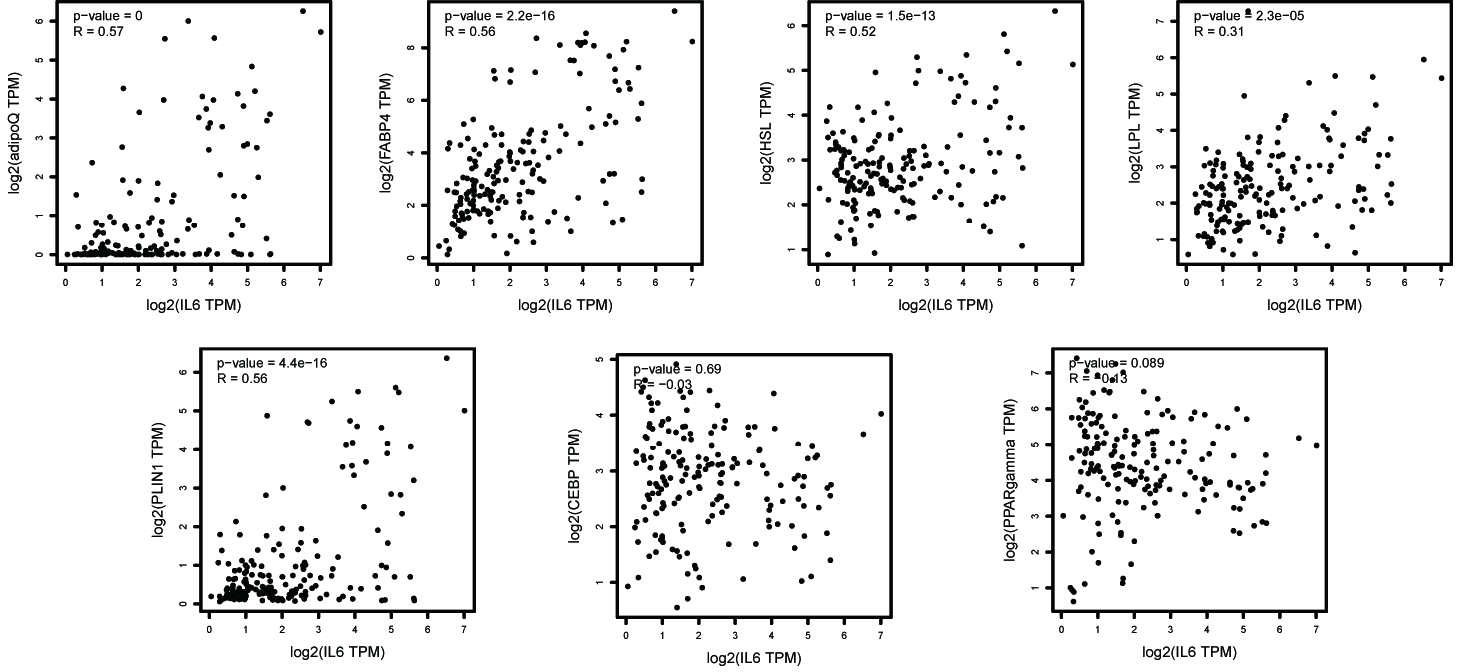

Supplement: Supplemental Material [file KADI_A_2084900_SM5599.zip › supplementary/Figure S2.tif]
